# Editorial: 4th Applied Synthetic Biology in Europe

**DOI:** 10.3389/fbioe.2020.00431

**Published:** 2020-05-06

**Authors:** Jean Marie François, Jussi Jantti, Fayza Daboussi

**Affiliations:** ^1^Toulouse Biotechnology Institute, Université de Toulouse, CNRS, INRA, INSA, Toulouse, France; ^2^Toulouse White Biotechnology Center, UMS-INSA-INRA-CNRS, Ramonville St Agnes, France; ^3^VTT Technical Research Centre of Finland, Espoo, Finland

**Keywords:** synthetic biology, genome engineering, metabolic engineering, evolution engineering, cell-free systems, bionanoengineering

The congress of Applied Synthetic Biology in Europe was the 4th of this kind to be organized under the auspices of the European Federation of Biotechnology in Toulouse from 24 to 27 October 2018. It gathered about 130 participants from all over Europe as well as some participants from Asia, Brazil, and USA. Five sessions encompassing main disciplines in Synthetic Biology were on the program, and we acknowledged receiving several contributions by many of the participants to this meeting in each of these sessions. The sessions that received the most contributions were those dealing with topics in synthetic pathways for bio (chemical) manufacturing and synthetic biology for biotechnology applications. Relevant in these reports were concerns about pathways design and reconstruction, sometimes guided by computational analysis to propose non-natural metabolic routes for maximal yield and the choice of the more appropriate microorganism as potential industrial producer. In this regard, the production of trans-cinnamate from glucose was achieved by rational engineering of L-phenylalanine accumulation in *Pseudomonas taiwensis* since this bacteria is much more tolerant than *E. coli* to this non-natural product (Otto et al.). However, toxicity of this product remains a serious bottleneck in order to reach economically viable titers and yields of *t-*cinnamate from sugars, which might be overcome by application of adaptive laboratory evolution. As a well-established microbial platform for fatty acid accumulation (Blazeck et al., 2015), the oleaginous *Yarrowia lipolytica* was engineered for *de novo* biosynthesis of odd fatty acid from inexpensive carbon source (Park et al.). The critical point in this work was to enable an endogenous supply of propionyl-CoA as the key precursor for the synthesis of these fatty acids. These authors brought this capability to the yeast cell by the expression of a modular pathway with seven enzymatic steps that employed the threonine biosynthetic route and its deamination into α-ketobutyrate. The industrial potential of this strategy will await further improvement in the specificity and activity of the decarboxylating enzyme on α-ketobutyrate as well as enhancement of carbon fluxes to propionyl-coA through metabolic engineering tools. Two other papers in this session were dedicated to implement non-natural pathways to produce bio-based products from C5 and C6 sugars at the highest yield. Bator et al. compared *in silico* and *in vivo* the synthetic capabilities of three metabolic routes, namely the xylose isomerase, the Dahms and the Weimberg pathways for xylose metabolization into a wide variety of valuable products in *Pseudomonas putida* KT2240. The computational analysis with flux balance analysis (FBA) indicated highest yield of 12 out of the 14 value products by the isomerase pathway, which was confirmed *in vivo* for the production of mono-rhamnolipids and pyocyanin. While reinforcing the usefulness of FBA to guide pathway design, the limitation of these *in silico* methods is that they cannot predict best productivity and titer. Ultimately, the work showed that higher production rate and titer were obtained with the Weimberg pathway for these high value products. Product yield higher than those calculated from the stoichiometry of natural pathways can be obtained by pathway reconstruction in which loss of carbon as CO_2_ is avoided, as recently shown for the construction of the “NOG” pathway (Bogorad et al., 2013) and reviewed by François et al.. Along this line, Lachaux et al. devised a cyclic pathway enabling optimal conversion of C5 and C6 carbon into glycolic acid by reallocating the function of two *E.coli kdsD* and *fsaA* genes encoding arabinose-5-P isomerase and fructose-6-P aldolase, respectively, in combination with the non-oxidative pentose pathway. Altogether, these contributions illustrate the trend of integrating systems biology, synthetic biology, and evolutionary engineering with traditional metabolic engineering to expedite the development of relevant microbial cell factories for the production of bio-based products from renewable carbon sources, as the cornerstone of the emerging Bio-Economy. However, there is still a gap to be filled in to make these strains industrially competitive and the attendant fermentation processes economically viable. Notably, better knowledge of the management of the cellular energy and redox balance by metabolic engineering as reviewed in Kalnenieks et al. for the ethanologenic bacterium *Zymomonas mobilis* and development of analytical tools for rapid screening of products yield, such as sensing and monitoring production of benzoic derivatives by mean of a biosensor (Castaño-Cerezo et al.) are important to make rapid progress in this applied field of Synthetic Biology.

Generation and investigation of biological functions can be in *in vitro* systems using cell-free systems that rests on the use of cell free protein synthesis (CFPS). Pioneered by Nirenberg and Matthaei (1961) to elucidate the genetic code, cell-free systems are gaining more and more credit in the field of Synthetic Biology as a prototype platform to engineer biological parts at levels of protein, metabolism and cell (Shimizu et al., 2006; Hodgman and Jewett, 2012; Moore et al., 2017). Cell free synthetic biology was particularly emphasized in the session “Biosciences and Bionanoengineering' with several contributions. Spice et al. reported on the synthesis and assembly of hepatitis B virus-like particles (VLP) using cell-free system prepared from *Pichia pastoris*, which can represent an alternative approach to the production of VLP for therapeutic purposes. On the other hand, Gabant and Borrero exploited cell-free protein synthesis system as a rapid method to expedite production and validation of a large set of bacteriocin, which are cataloged into a database, termed “PARAGEN1.0.” Cell free system is also suggested as an alternative strategy for engineering and production of organophosphorus hydrolysing enzymes to be used to decontaminate highly toxic agricultural pesticides and chemical warfare (Thakur et al.). In addition, these authors make a thorough overview on the practice that are currently used for decontaminating environments and seawater from toxic organophosphorus compounds, emphasizing on the need to strongly enhance catalytic activity and stability of these organophosphorus hydrolysing enzymes by various bionanoengineering approaches.

Genome editing, genome engineering, and synthetic genomics are among the most disruptive technology at the heart of Synthetic Biology. A contribution to this session was a paper by Millacura et al. who report on a cell-based logic system termed ParAlleL that allows to construct complex logic circuits enable to respond to a combination of inputs. They demonstrate their concept through the construction of three simple logic circuits, a 1-bit full adder, a 1-bit subtractor, and a numeric display. Experimentally, the concept was shown by transforming *E. coli* with three out of six plasmids to make them able to survive only when precisely three antibiotics are provided. This represents an interesting way of constructing circuits from cellular populations in which logic is constructed and circuitry functionality displayed simply by the surviving cells in the presence of antibiotics. Very challenging in genetic and genome engineering is to build efficient and robust gene expression leading to high protein production, for instance. Eichmann et al. tentatively solved the problem of expression and production of so-called three difficult-to-express proteins by systematic evaluation of ribosome binding sites, N-terminal affinity tags and periplasmic translocation sequences to which was fused green fluorescent protein (GFP) to identify high producers by fluorescence activated cell screening (FACS). Large combinatorial libraries of genes were expressed and the outcome was measured in a platform monitoring expression automatically. Though yields were still low, this work unraveled that TAT-dependent signal sequence from *E. coli* YahJ for expression is recognized in the fast-growing bacterium *Vibrio natriegens*, which could be used as an industrial platform for production of recombinant proteins.

This editorial can be concluded by referring to the article by El Karoui et al. which reports on a workshop that brought together British academics and industrialists at the University of Edinburgh to discuss the future of synthetic biology. If processes that underlie all living systems can be manipulated, modified or reshaped using the tools of Synthetic Biology, then in return we need to take the public with us on this journey to create a productive dialogue about the work we can do and the impact it can have on our world and our way of life.

## Author Contributions

All authors listed have made a substantial, direct and intellectual contribution to the work, and approved it for publication.

## Conflict of Interest

JJ was employed by company VTT Technical Research Centre of Finland. The remaining authors declare that the research was conducted in the absence of any commercial or financial relationships that could be construed as a potential conflict of interest.
